# Molecular biomarker responses in the freshwater mussel *Anodonta anatina* exposed to an industrial wastewater effluent

**DOI:** 10.1007/s11356-021-15633-4

**Published:** 2021-08-07

**Authors:** Gustaf MO Ekelund Ugge, Annie Jonsson, Olof Berglund

**Affiliations:** 1 Department of Biology, Lund University, Sölvegatan 37, 223 62 Lund, Sweden; 2 School of Bioscience, University of Skövde, Högskolevägen 3, 541 46 Skövde, Sweden

**Keywords:** Bivalve, Effect size, Mixture toxicity, RT-qPCR, Sex effects, Wastewater

## Abstract

Using a selection of molecular biomarkers, we evaluated responses in freshwater mussels (*Anodonta anatina*) exposed to effluent from an industrial wastewater treatment facility. The aims of this work were to (1) assess biomarkers of general toxicity under sublethal exposure to an anthropogenic mixture of chemicals, represented by an arbitrary effluent, and (2) evaluate the potential of *A. anatina* as a bioindicator of pollution. Adult mussels (*n =* in total 32; 24 males and 8 females) were exposed (96 h) in the laboratory to a fixed dilution of effluent or to a control treatment of standardized freshwater. Metal concentrations were in general higher in the effluent, by an order of magnitude or more, compared to the control. Toxic unit estimates were used as proxies of chemical stress, and Cu, Ni, and Zn were identified as potential major contributors (Cu> Ni > Zn). Six transcriptional (*cat*, *gst*, *hsp70*, *hsp90*, *mt*, *sod*) and two biochemical (AChE, GST) biomarkers were analyzed in two tissues, gills, and digestive glands. Out of the 16 responses (eight biomarkers × two tissues), 14 effect sizes were small (within ± 28 % of control) and differences non-significant (*p* > 0.05). Results did however show that (1) AChE activity increased by 40% in gills of exposed mussels compared to control, (2) *hsp90* expression was 100% higher in exposed female gills compared to control, and (3) three marker signals (AChE in both tissues, and *hsp70* in gills) differed between sexes, independent of treatment. Results highlight a need for further investigation of molecular biomarker variability and robustness in *A. anatina*.

**Supplementary Information:**

The online version contains supplementary material available at 10.1007/s11356-021-15633-4.

## Introduction

Chemically complex pollution from anthropogenic activities is a major concern in environmental protection and has gained considerable attention in ecotoxicology and environmental sciences. As a result of daily use in human activities, for instance, agriculture, industrial production, and use of, e.g., pharmaceuticals and personal care products, a variety of natural and synthetic compounds may eventually enter the environment (Anliker et al. 2020; Herrero-Hernández et al. 2020; Su et al. 2020; Vareda et al. 2019). Industries and households, via wastewater effluents or runoff, constitute major sources of complex pollution to aquatic recipients (Chen et al. 2020; Ellis and Butler 2015; López-Pacheco et al. 2019). Although separate pollutants in, e.g., treated wastewater often occur at low concentrations (Farkas et al. 2020; Vareda et al. 2019; Wang et al. 2018), they may interact and contribute to additive or synergistic biological effects when in mixtures, causing adverse effects in exposed organisms (Aronzon et al. 2020; Cedergreen 2014; Mebane et al. 2020; Wang et al. 2019). Therefore, in anthropogenic mixtures, such as wastewater effluents, with few conspicuous chemical parameters or without prior knowledge of the chemical composition, general biomarkers of chemical stress might be useful for detection of sublethal mixture toxicity. Furthermore, early biomarker responses can potentially be used under both laboratory and field settings to anticipate harmful effects from pollutant exposure and may, in the long term, improve strategies of monitoring of sensitive ecosystems and protection of recipients (e.g., van der Oost et al. 2003).

Potential responses in organisms under toxic exposures include changes in molecular parameters, such as enzyme activity or transcript levels, some of which are commonly used as biomarkers to detect general chemical stress (Lehtonen et al. 2016; Perić and Burić 2019; Tsangaris et al. 2016). By definition, biomarkers are used to detect deviations from a normal state (e.g., van der Oost et al. 2003), which is often defined by a control group. However, there is a lack of data describing variabilities in responses and baseline signals, making it difficult to distinguish stress responses from background noise, i.e., normal variation. For molecular markers, it is often unclear how response magnitudes (i.e., effect sizes) vary with interacting internal (e.g., tissue, sex) and external factors (for instance, chemical composition, toxicant concentration, exposure time) (e.g., Bahamonde et al. 2016).

In this study, we assessed biochemical and transcriptional responses that represent commonly used biomarkers of general toxicity and chemical stress. For instance, the enzyme acetylcholinesterase (AChE) is involved in neurosignaling, and its activity in bivalves may respond to different types of mixture exposures (e.g., Aguirre-Martínez and Martín-Díaz 2020; Perić and Burić 2019; Tsangaris et al. 2016). Heat shock proteins protect cellular integrity and respond to a wide range of both chemical and physical stressors (Ferreira-Rodríguez et al. 2018; Liu et al. 2014, 2016). Catalase (CAT) and superoxide dismutase (SOD) protect against oxidative stress, and their activities as well as transcript levels may respond to mixture exposure (Bigot et al. 2011; Gonzalez-Rey et al. 2014; Lehtonen et al. 2016; Turja et al. 2013). Metallothionein (MT) is involved in maintaining cellular metal homeostasis and responds to various metal stressors (Bigot et al. 2011; Mourgaud et al. 2002), and glutathione-S-transferase (GST) is an enzyme important in toxicant metabolism and detoxification, responding to various stressors and mixtures (Bigot et al. 2011; Lehtonen et al. 2016; Perić and Burić 2019; Turja et al. 2013). Using the freshwater duck mussel (*Anodonta anatina*), we measured enzyme activities of AChE and GST, while *cat*, *gst*, heat shock protein 70 (*hsp70*), heat shock protein 90 (*hsp90*), *mt*, and *sod* were measured on the transcriptional level.

*A. anatina* is native to and widely distributed in Scandinavian and many European freshwater ecosystems (Lopes-Lima 2014). Bivalves are likely exposed to toxicants occurring in their (natural or laboratory) environment due to sessility and filtration feeding, and *A. anatina* could serve as an ecologically relevant freshwater model in ecotoxicology. Previous studies cover, e.g., pollutant uptake (Berglund et al. 2019; Nugroho and Frank 2011), molecular and behavioral biomarkers (Bielen et al. 2016; Falfushynska et al. 2013; Hartmann et al. 2016; Oliviera et al. 2015), and mortality (Kováts et al. 2010; Oliviera et al. 2015). The reproductive cycle of *A. anatina* includes a gravid stage during autumn/winter (Aldridge 1999; Hinzmann et al. 2013), potentially increasing variability in transcriptional and biochemical biomarkers (Ekelund Ugge et al. 2020).

Our objectives were to (1) evaluate selected responses as biomarkers of sublethal exposure to chemically complex, anthropogenic pollution and (2) assess *A. anatina* as a bioindicator species. An industrial wastewater effluent, i.e., a complex mixture, was used to represent an arbitrary anthropogenic stressor. While constituting a mixture of organic and inorganic substances, the main focus was, for practical reasons, limited to evaluation of metals. The selected biomarkers were assessed in *A. anatina* after acute (96 h) laboratory exposure to either a single effluent concentration or a control treatment of standardized freshwater. We hypothesized that (1) biomarker signals in digestive glands and gills would differ between effluent exposed and non-exposed mussels and that (2) gravid mussels would show different baseline signals (fixed effects) and/or response magnitudes (treatment interactions) compared to non-gravid.

## Material and methods

### Mussel collection and maintenance

Adult mussels (length 92 ± 17 mm) were collected on September 19, 2018, in Vinne å (Southern Sweden, 56° 06′45″ N, 13° 54′35″ E). The location is adjacent to human settlement and subject to, e.g., recreational fishing, but free from point sources of pollution. After being brought to the laboratory, the mussels were acclimatized to laboratory conditions for 26 days. During this period, a preliminary range-finding experiment was performed on a different subset of mussels (details presented in appendix A). Acclimatizing mussels were kept in two 40 L aquaria containing 30 L continuously aerated standardized freshwater (ISO 6341: 2012) with a nominal water hardness of 250 mg/L CaO_3_. As bottom substrate, each aquarium contained an approximately 5 cm sand layer (0.2–0.7 mm grain size). Standardized freshwater and bottom substrate were both prepared as previously reported (Ekelund Ugge et al. 2020). Three times weekly, 15–20 L medium was renewed, and main experiment mussels were randomly re-distributed between aquaria to avoid tank effects on acclimatization. Additions of *Pseudokirchneriella subcapitata* were made to feed the mussels, corresponding to approximately 3.2 × 10^6^ cells × mussel^-1^ × day^-1^. No food was added within 48 h prior to the start of the experiment. During acclimatization and experimental periods, water temperature was 21±1° C, and the light cycle was 16 h light: 8 h dark.

### Experimental treatment

Frozen samples of treated effluent water were obtained from an industrial wastewater treatment facility. The facility receives process and sanitary wastewater, as well as runoff, from an industrial area where mainly organic chemical products are manufactured. Compounds such as organic acids, phenols, and aldehydes may occur at high concentrations in incoming wastewater but are efficiently removed in the treatment process (personal communication). Phenol and aldehyde samples from the sampling period measured <0.05 mg/L and <0.5 mg/L, respectively, as reported by the industry. Other plausible organic contaminants from raw materials and manufactured products have previously been analyzed but not detected and are therefore not routinely monitored. The industry reported an effluent total organic carbon (TOC) content ranging between 31 and 36 mg/L for the period when water was sampled (median 33 mg/L). Based on previous evaluation, this is assumed to largely consist of non-toxic microbial degradation products (personal communication). Organic pollutants were therefore not measured in this study, and instead, metal contamination was selected as the main focus and used as a proxy to represent the chemical complexity. Effluent metal concentrations are continuously monitored by the industry, and contamination is believed to result mainly from corrosion and erosion of, e.g., metal piping, galvanized steel, and stainless steel equipment, in the processing of organic compounds. A minor fraction is believed to result directly from manufacturing of metal containing products, and in addition, there might be metal contamination from raw materials and runoff from loading areas (personal communication).

Eight effluent samples, representing 8 consecutive days of effluents from the treatment facility, were thawed and mixed flow proportionally. During exposure, mussels were kept individually in glass containers (Ø 12 cm) of 1 L aerated medium (effluent or standardized freshwater), with 0.3 L sand added as bottom substrate. Light, temperature, and feeding conditions were the same as during acclimatization. Acute exposures of 96 h were performed as a trade-off between capturing immediate responses and allowing for potential time-dependent uptake of pollutants. After 96 ± 0.5 h, mussels were dissected. Gravid mussels were distinguished visually by the presence of immature glochidia in the gills (Figure A.1, appendix A). Gill and digestive gland tissues were dissected, and subsamples were immediately snap frozen in liquid nitrogen and subsequently stored at −80°C for biochemical assays and chemical analysis or submerged in RNA-Later (Invitrogen, USA) and stored at −20°C for gene expression analyses.

A preliminary range-finding experiment was performed to select an effluent concentration for the main exposure experiment (details presented in appendix A). A dilution to 60% of the initial concentration was selected, as this was the highest test concentration that did not appear to impair mussel filtration (roughly estimated by daily visual inspection of valve opening). In addition, this exposure, although overlapping with control treatment variation and not being replicated, showed implication of AChE inhibition (Figure A.2, appendix A).

In the main experiment, mussels were exposed to 60% industrial effluent water (*n =* 16) or a control treatment of standardized freshwater (*n =* 16). Glass containers with exposure media were prepared and kept under aeration approximately 24 h prior to experimental start. Before the addition of mussels, each container was sampled for chemical analysis of exposure media. pH and oxygen were monitored at 0 h, 48 h, and 96 h. Initial pH was 7.8 (± 0.04) and 8.2 (± 0.03) in control and effluent treatments respectively, steadily decreasing to 7.1 (± 0.3) and 7.7 (± 0.2) after 96 h. Oxygen saturation was consistently ≥90 % (7.9–9.1 mg O_2_/L) in all containers, except for one effluent container in which saturation was 80 % (7.1 mg O_2_/L) at 48 h. After the experiment was ended, distribution of gravid and non-gravid mussels was determined to be 3:13 and 5:11 in the control and effluent treatment, respectively.

### Chemical analysis

A number of elements were analyzed in exposure media sampled (non-filtered) at the experimental start (Table 1) and in water samples from Vinne å (Table A.1, appendix A). Samples were kept frozen (−20°C) prior to analysis, and one control treatment sample was lost during freezing. Upon arrival to the chemical analysis laboratory, the water samples were acidified by addition of nitric acid (1% v/v), then analyzed by inductively coupled plasma sector field mass spectrometry (ICP-SFMS) (*Element*, Thermo Scientific, Germany), inductively coupled plasma atomic emission spectrometry (ICP-AES) (*Agilent ICP-OES 725*, Agilent, USA), and atomic fluorescence spectrometry (AFS) (*PSA Millennium Merlin*, P S Analytical, UK) according to standards from the International Organization for Standardization and the US Environmental Protection Agency (ISO 17852:^2006^, 11885:^2007^, 17294-2:^2016^. U.S. EPA 1994a, 1994b). Metal content was also determined in snap frozen tissue samples remaining after biochemical assays. Tissues were subject to nitric acid/hydrogen peroxide digestion, and metals were analyzed by ICP-SFMS (*Element 2*, Thermo Scientific, Germany) (ISO 17294-2: 2016; U.S. EPA 1994b) (Table 1).

**Table. 1:** Median concentration (min-max) of elements measured in in water and tissue samples (*n* = 16 per group).

		**Ca**	**K**	**Mg**	**Na**	**Al**	**As**	**Ba**	**Cd**
Water (μg/L)	Control*	77 000 (74 000–81 000)	3 200 (3 000–3 500)	10 000 (10 000–11 000)	19 000 (18 000–21 000)	25 (13–57)	0.061 (0.050^†^–0.094)	53 (25–63)	0.0052 (0.0020^†^–0.014)
	Effluent	40 000 (36 000–42 000)	14 000 (10 000–15 000)	6 000 (4 400–6 100)	370 000 (330 000–380 000)	48 (38–97)	0.23 (0.15–0.36)	82 (57–110)	0.023 (0.016–0.033)
Dig. gland (mg/kg WW)	Control	290 (130–610)	410 (370–680)	NA	230 (180–360)	NA	1.1 (0.49–1.3)	NA	0.056 (0.035–0.089)
	Effluent	260 (110–520)	480 (360–1 500)	NA	310 (230–560)	NA	1.0 (0.56–1.3)	NA	0.056 (0.028–0.095)
Gills (mg/kg WW)	Control	35 000 (12 000–71 000)	510 (360–750)	NA	420 (240–1 300)	NA	0.89 (0.30–2.4)	NA	0.14 (0.023–0.29)
	Effluent	31 000 (11 000–71 000)	550 (170–1 100)	NA	530 (200–670)	NA	0.89 (0.35–1.9)	NA	0.15 (0.042–0.25)
		**Co**	**Cr**	**Cu**	**Fe**	**Hg**	**Mn**	**Mo**	
Water (μg/L)	Control*	0.027 (0.0059–0.072)	0.023 (0.010^†^–0.054)	0.68 (0.48–0.89)	4.5 (0.59–41)	<0.0020^‡^	2.5 (1.5–5.9)	0.27 (0.17–0.33)	
	Effluent	0.21 (0.17–0.33)	3.1 (2.3–3.7)	7.7 (5.1–13)	28 (16–54)	<0.0020^‡^	6.5 (2.8–9.2)	9.1 (7.9–9.5)	
Dig. gland (mg/kg WW)	Control	0.078 (0.045–0.28)	0.032 (0.030^†^–0.070)	3.2 (2.3–4.5)	73 (42–120)	0.027^†^ (0.014–0.033)	14 (1.8–40)	NA	
	Effluent	0.079 (0.058–0.20)	0.040 (0.030^†^–0.13)	3.2 (2.2–5.2)	69 (27–120)	0.023 (0.011–0.051)	10 (1.8–35)	NA	
Gills (mg/kg WW)	Control	0.17 (0.033–1.0)	0.26 (0.048–0.55)	1.3 (0.71–3.4)	470 (89–1500)	0.013 (0.010^†^–0.023)	2 300 (500–5 600)	NA	
	Effluent	0.19 (0.052–0.62)	0.23 (0.11–0.76)	1.3 (0.83–8.6)	510 (120–1800)	0.013 (0.010^†^–0.017)	2 200 (1 000–5 300)	NA	
		**Ni**	**P**	**Pb**	**Si**	**Sr**	**V**	**Zn**	
Water (μg/L)	Control*	0.061 (0.050^†^–0.085)	7.2 (3.4–14)	0.028 (0.010^†^–0.061)	960 (600–1 300)	68 (50–80)	0.15 (0.075–0.22)	0.54 (0.34–1.4)	
	Effluent	16 (12–19)	120 (93–140)	0.11 (0.038–0.18)	2 200 (2 000–2 300)	69 (58–77)	0.43 (0.33–0.63)	8.4 (4.4–18)	
Dig. gland (mg/kg WW)	Control	0.073 (0.053–0.12)	NA	0.040^†^ (0.040–0.10)	NA	NA	NA	12 (8.4–15)	
	Effluent	0.13 (0.072–0.29)	NA	0.040^†^ (0.040–0.20)	NA	NA	NA	12 (8.8–14)	
Gills (mg/kg WW)	Control	0.078 (0.040^†^–0.18)	NA	0.055 (0.040^†^–0.11)	NA	NA	NA	130 (24–290)	
	Effluent	0.10 (0.040^†^–0.26)	NA	0.058 (0.040^†^–21)	NA	NA	NA	130 (36–290)	

*n = 15

^†^≥ 1 sample <LOR, assumed to equal LOR

^‡^All samples <LOR

### Estimation of chemical stress

Toxic units (TUs) were calculated for sublethal organism effects (behavioral/ growth/ physiological/cellular/biochemical endpoints) and mortality, respectively (TU EC_50_ and TU LC_50_), to quantitatively estimate the chemical stress imposed by effluent exposure. TUs were determined for elements measured in water, a priori excluding the non-metals P and Si as well as macrominerals Ca, K, Na, and Mg. Elements for which toxicity data was not found were simply reported as “not applicable” (NA) (Table A.2, appendix A). Mollusk 96-h 50% effect concentrations (EC_50_) and 96-h 50% lethal concentrations (LC_50_) from laboratory experiments were retrieved from the US EPA ECOTOX database (https://cfpub.epa.gov/ecotox/) on September 7–8, 2020 (details presented in appendices B and C, deposited at https://data.mendeley.com/datasets/jc469bc5mv/1). Toxic units (TU EC_50_ and TU LC_50_) were calculated for each metal as TU XC_50_ = Measured conc. /Mollusk 96 h XC_50_, with measured concentration and effect concentration represented by the respective median. TUs for all metals were added, as *TU*_*M*_*XC*_50_ =  ∑ *TU XC*_50_, to represent additive mixture toxicity (TU_M_ EC_50_ and TU_M_ LC_50_) for each treatment (Table A.2, appendix A).

As an additional estimate of relative contributions to stress, measured metal concentrations were, when applicable, converted to fraction of the respective environmental quality standards (EQSs) for inland surface waters, as Fraction of EQS = Measured conc. /EQS. Measured concentrations were represented by the median of measured total concentration, and EQSs by the respective annual mean EQS from European and Swedish legislation (European Parliament and Council 2013; Havs- och vattenmyndigheten 2019) (Table A.2, appendix A).

### Biomarker assays

The biomarker selection consisted of eight molecular biomarkers. The enzymatic assays for AChE and GST activities were based on the rates for hydrolysis of acetylthiocholine (Bocquené and Galgani 1998) and glutathione conjugation to 1-chloro-2,4-dinitrobenzene (Habig et al. 1974), respectively. Samples were prepared in phosphate buffers, and spectrophotometric analyses (using a *SpectraMax 190* plate reader (Molecular Devices, USA)) were performed, all according to previous description (Ekelund Ugge et al. 2020). All enzyme activities were normalized, first by sample protein concentration (Bradford 1976) and second to the mean activity of control samples of the respective tissue.

For transcriptional markers (*cat*, *gst*, *hsp70*, *hsp90*, *mt*, and *sod*), relative transcript levels were measured by reverse transcription quantitative polymerase chain reaction (RT-qPCR). RNA was extracted using the *Norgen’s Total RNA Purification Kit* (Norgen, Canada), including 40 μM of DL-dithiothreitol (DTT, Promega, USA) in the lysis buffer. Tissues were homogenized using a *TissueLyser II* (Qiagen, Germany) and 5-mm stainless steel beads (Qiagen, Germany). RNA amounts and A260/A280 ratios (showing 1.9–2.1) were measured using a *NanoDrop 2000* spectrophotometer (Thermo Scientific, USA). Before cDNA synthesis, the RNA was DNAse treated using the *Heat&Run gDNA removal kit* according to instructions (ArcticZymes, Norway), and the RNA integrity was assessed in randomly selected subset of samples using a *Fragment Analyzer* (Advanced Analytical, Austria). Since our interest was relative rather than absolute gene expression, and only short sequences were targeted (<200 bp, Ekelund Ugge et al. 2020, appendix), reverse transcription was performed despite apparent RNA degradation (RNA quality numbers (RQN) of 1.8–2.3 and 2.8–4.5 in the gills and digestive glands, respectively). cDNA was synthesized by reverse transcription of 200 ng and 100 ng RNA for digestive gland and gills, respectively, using the *TATAA GrandScript cDNA* synthesis kit (TATAA Biocenter AB, Sweden). The qPCR assays were performed as previously described (primer sequences are presented in Table A.3, appendix A, and assay details are found in Ekelund Ugge et al. 2020). Gene expression was determined by the 2^-ΔΔCt^ method (Livak and Schmittgen 2001), where expressions were normalized by the mean of control samples of the gill and digestive gland tissue, respectively, and then internally for each individual sample by the mean of two reference genes, *β-actin* and *28S* rRNA.

### Statistics

Statistical analyses were run and figures were generated in R version 4.0.2 (R Core Team 2020). For the principal component analysis (PCA), water and tissue concentrations of measured metals were normalized as percentage of the respective mean from control samples and log_10_-transformed. For various chemical parameters, there were samples showing concentrations below the levels of reporting (LOR). Unless that was true for the majority (≥ 50 %) of samples, concentrations <LOR were assumed to equal the respective LOR. If, however, a majority of samples displayed concentrations <LOR, as for Hg in water and Pb in digestive glands, the parameter was removed completely from the respective PCA. For elements measured in both tissue and water samples, correlations (Pearson) were determined for measured concentrations (log_10_-transformed), excluding datapoints for which either concentration (tissue or water) was <LOR. Biomarker responses (log_2_-transformed) were analyzed by linear models and separated by tissue. Treatment, sex, and the treatment/sex interaction were used as fixed factors in the full models. Model selection for linear models was based on lowest AIC scores after sequential ANOVA analysis, where least significant (*p>*0.05) effect factors were removed one at a time. Residual normality for biomarker responses was assessed by Shapiro-Wilk normality tests and Q-Q plots. Significant differences (*α* = 0.05) in the final models were identified with a Tukey HSD post hoc test, using the “emmeans” package (Lenth 2020). Treatment effects on overall variation were assessed by a paired *t*-test, in which the coefficient of variation (CV) of each biomarker/tissue pair was compared between control and exposed mussels. CV was calculated by dividing each biomarker/tissue standard deviation by corresponding mean response (non-log-transformed). Finally, the “ggplot2” package (Wickham 2016) was used to produce all figures.

## Results

**Figure 1:**
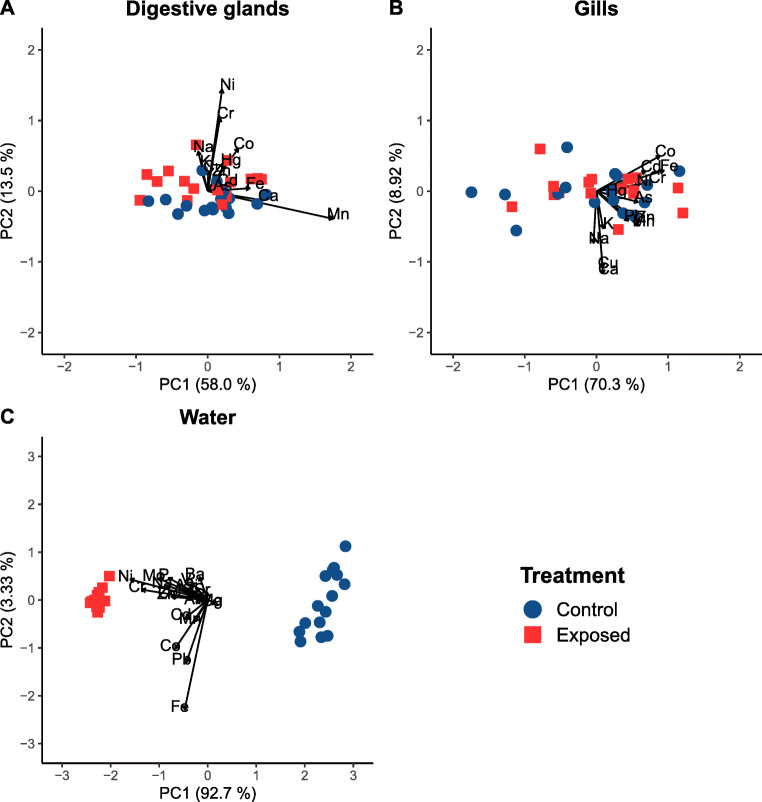
Principal component analyses of metal content (as well as P and Si content in water) in **A** digestive glands and **B** gills of *Anodonta anatina* after 96-h exposure to industrial wastewater effluent and **C** initial concentrations in water. Arrows show relative weights of each measured parameter and are scaled to the length of the plot axes

With regards to chemical composition, digestive gland control and effluent samples were slightly separated along principal component 2 of the PCA (Figure 1A), while there was no apparent difference in gills (Figure 1B). The control and effluent exposure media were however highly separated along principal component 1 (Figure 1C). Most concentrations were higher in the effluent medium than in the control medium, many by an order of magnitude (e.g., Na, Co, Cu, Fe, Mo, P, and Zn) or more (e.g., Cr and Ni) (Table 1). The only elements at higher concentration in the control medium were Ca and Mg, with roughly twice as high concentrations as in the effluent exposure. In contrast, tissue levels were overall similar between treatments (Table 1). The most notable difference was an approximately 2-fold higher digestive gland concentration of Ni in exposed mussels compared to control, positively correlated to exposure concentration (Table 1, Figure A.3, Table A.4, appendix A). In addition, digestive gland Na levels were also positively correlated to exposure concentration, while no correlation was detected between tissue and water concentrations of As, Ca, Cd, Co, Cr, Cu, Fe, K, Mn, Pb, and Zn (Figure A.3, Table A.4, appendix A).

The effluent and control TU_M_ EC_50_ were 0.15 and 0.020, respectively (Table A.2, appendix A). This corresponds to approximately 1/7 and 1/50 of estimated median effect concentration for sublethal organism effects to occur. With regards to mortality, the effluent TU_M_ LC_50_ was 0.039 (1/26 of the estimated median lethal metal exposure), while the control TU_M_ LC_50_ was 0.0028 (1/360 of the median lethal exposure) (Table A.2, appendix A). Based on TU estimations, Cu contributed the most to metal toxicity, followed by Ni, Zn, and Al (Al>Zn for sublethal organism effects, Zn>Al for mortality). Finally, total concentrations of Cu, Ni, and Zn in the effluent exposure exceeded their respective EQS for bioavailable concentration in inland surface waters, by factors of 15, 4.1, and 1.5, respectively, while Cu in the control exceeded the EQS by a factor of 1.4 (Table A.2, appendix A).

**Figure 2:**
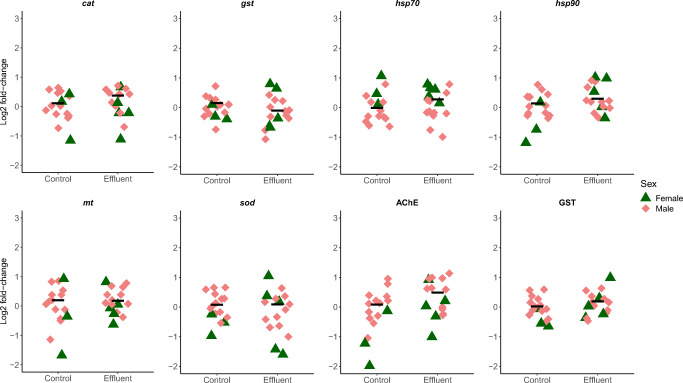
Biomarker responses (log_2_ fold change relative control) in gills of Anodonta anatina exposed to a control treatment (n= 16) or an industrial wastewater effluent (n = 16) for 96 h. Bars correspond to median responses

**Figure 3:**
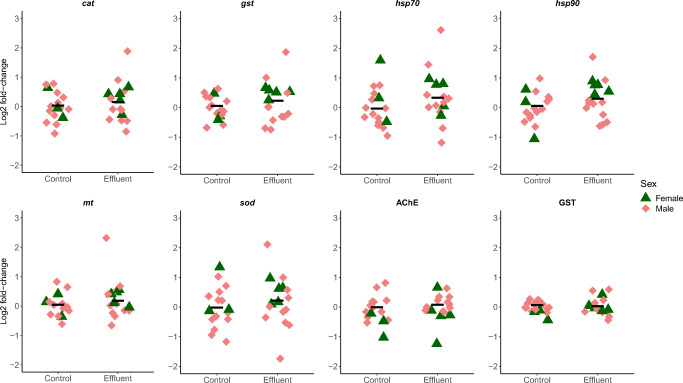
Biomarker responses (log_2_ fold change relative control) in digestive glands of Anodonta anatina exposed to a control treatment (n= 16) or an industrial wastewater effluent (n= 16) for 96 h. Bars correspond to median responses

Only two of the eight biomarkers, AChE and *hsp90*, showed a significant treatment effect and interaction, respectively, and only in gills. Other biomarker responses to the effluent exposure were within ± 28 % of the respective control (log_2_ fold changes ranging from −0.19 to 0.36), across both gills and digestive glands (Table 2, Figures 2 and 3). AChE activity in exposed mussels was 40% higher than control (log_2_ fold change = 0.48) (Table 2, Figure 2). For *hsp90*, a treatment/sex interaction revealed a higher expression in both exposed females (100% higher, log_2_ fold change = 1.0) and males (79 % higher, log_2_ fold change = 0.84) compared to control females (Table 2, Figure 2).

**Table 2:** Final model for biomarker endpoints and effect sizes of treatment responses, sex differences and treatment/sex interactions. F statistics, degrees of freedom, model term *p* values and significant differences (post hoc) are presented for the linear models. Mean treatment responses are presented for all biomarkers except when there was a significant treatment/sex interaction. Mean sex differences and treatment/sex interactions are presented when included in the final model

Endpoint		Final model	Factor	F	d.f.	*p*	Significant differences (post hoc)	Treatment response (log_2_ fold change in WW compared to C)	Sex difference (log_2_ fold change in M compared to F)	Treatment/sex interaction (log_2_ fold-change compared to mean C)
Dig. gland	*cat*	Resp ~ 1	–	–	0, 31	–	–	0.157	–	–
	*gst*	Resp ~ 1	–	–	0, 31	–	–	0.205	–	–
	*hsp70*	Resp ~ 1	–	–	0, 31	–	–	0.357	–	–
	*hsp90*	Resp ~ Treat	Treat	2.17	1, 30	0.151	–	0.298	–	–
	*mt*	Resp ~ 1	–	–	0, 31	–	–	0.244	–	–
	*sod*	Resp ~ Sex	Sex	2.44	1, 30	0.129	–	0.191	–0.480	–
	AChE	Resp ~ Sex	Sex	7.69	1, 30	0.00944	**F<M** (*p =* 0.00944)	0.0736	**0.456**	–
	GST	Resp ~ 1	–	–	0, 31	–	–	0.0376	–	–
Gills	*cat*	Resp ~ Sex	Sex	2.33	1, 30	0.137	–	0.137	0.311	–
	*gst*	Resp ~ 1	–	–	0, 31	–	–	–0.142	–	–
	*hsp70*	Resp ~ Sex	Sex	13.3	1, 30	0.000983	**F>M** (*p =* 0.000983)	0.104	**–0.634**	**–**
	*hsp90*	Resp ~	Treat	3.84	1, 28	0.0600	**C:F<WW:F** (*p =* 0.0222)	–	–	**–0.581 (C:F)**^a^
		Treat ×	Sex	1.01	1, 28	0.323	**C:F<WW:M** (*p =* 0.0396)			0.134 (C:M)^a, b^
		Sex	Treat/Sex	5.61	1, 28	0.0249				**0.442 (WW:F)**^b^
										**0.257 (WW:M)**^b^
	*mt*	Resp ~ 1	–	–	0, 31	–	–	0.165	–	–
	*sod*	Resp ~ Sex	Sex	2.66	1, 30	0.114	–	–0.189	0.399	–
	AChE	Resp ~ Treat +	Treat	5.09	1, 29	0.0317	**C<WW** (*p =* 0.0118)	**0.484**		**–**
		Sex	Sex	9.88	1, 29	0.00384	**F<M** (*p =* 0.00384)		**0.690**	
	GST	Resp ~ 1	–	–	0, 31	–	–	0.158	–	–

*F* females, *M* males, *C* control, *WW* wastewater effluent. For treatment/sex interactions, significant differences (*p*<0.05) are indicated by different letters

Two gills and one digestive gland biomarker showed sex differences, independent of treatment. AChE activity and *hsp70* expression were 61 % higher and 36 % lower, respectively, in male gills compared to females (log_2_ fold changes = 0.69 and −0.63, respectively, Table 2, Figure 2). In digestive glands, males demonstrated a 37% higher baseline AChE activity than females (log_2_ fold change = 0.46, Table 2, Figure 3).

Biomarker CVs ranged from 13 to 85% (Figure A.4, appendix A). Assessed pairwise across biomarkers and tissues; variation increased with effluent exposure in eleven out of 16 biomarker/tissue pairs, and decreased in five (Figure A.4, appendix A). The median CV increased from 32 in control treatments to 38 % in the effluent group (*p =* 0.017, Figure A.4, appendix A).

## Discussion

### Exposure and chemical stress

The selected industry mainly produces organic chemical products (personal communication). Consistently, monitoring data from the industry showed TOC levels around 30 mg/L in the undiluted effluent, and we assumed elevated TOC for effluent exposures relative the control. By comparison, our daily feeding of the mussels would have added a negligible amount of up to approximately 100 μg organic carbon L^−1^ day^−1^ in each exposure tank, assuming an algal carbon content in the range of 5–30 pg/cell (e.g., Pérez-Morales et al. 2015). In routine monitoring performed by the industry, phenol and aldehyde levels in the effluent were typically below detection limits, and when analyzed, other plausible organic pollutants have not been detected. Therefore, without dismissing potential impact from or interactions with organic and other inorganic substances, the focus of this study was, however, narrowed down to metal toxicity, as a proxy of chemical stress from mixture toxicity.

The effluent metal content was in general, when adjusted for dilution, within the orders of magnitude previously reported by the industry (personal communication). The exceptions were Ca and Mg, both occurring at approximately twice as high concentration in the control as in the exposure medium. As essential components in the standardized freshwater, they are assumed non-toxic at current concentrations. Remaining metals occurred at higher concentration compared to the control treatment, suggesting that the effluent exposure might, by comparison, be viewed as a complex chemical stressor, even without considering the potential contribution from organic or other inorganic compounds.

Effluent TU_M_ EC_50_ and TU_M_ LC_50_ were both an order of magnitude higher than the control, further implying a higher level of chemical stress. Three of the metals contributing most to toxicity, Cu, Ni, and Zn, showed levels at least an order of magnitude higher as compared to the control. Total concentrations of these metals exceeded their respective EQS for inland surface waters, although environmental impact assessment is to be based specifically on the bioavailable fraction. For instance, metal bioavailability can decrease with, e.g., water hardness and dissolved organic carbon (Bourgeault et al. 2010; Shoults-Wilson et al. 2010; Wang et al. 2009), implying bioavailability below 100% of the total concentration under current settings. On the other hand, effluent concentrations of Cu, Ni, and Zn, but also e.g. Cr, were all within ranges that separately may trigger various molecular responses in bivalves upon acute (72–96 h) exposures (e.g., Ciacci et al. 2012; Franzellitti et al. 2020; Li et al. 2018; Potet et al. 2016). We therefore argue that the current effluent exposure represents a sublethal acute stressor to which molecular responses, albeit not whole-organism effects, would be expected.

Metal uptake in bivalve soft tissues may be observable within hours (e.g., Cai and Wang 2019; Lee and Lee 2005), suggesting that 96-h exposure would be sufficient for uptake to occur. Yet, apart from Ni and Na in digestive glands, we could detect no correlation between external exposure and tissue concentration. The body burden depends on uptake from water, dietary uptake, and elimination rates, all of which are variable (Luoma and Rainbow 2005), and even with sufficient time, uptake might be limited by bioavailability. On the other hand, dietary sources might contribute substantially to total metal uptake (Lee et al. 2015; Luoma and Rainbow 2005). Filter feeding would thus be a potential exposure route of metals associated with algal cells or present in particulate forms (Hull et al. 2013; Lee et al. 2015). Considering the static exposure and moderate concentrations of each separate metal, elimination rates after 96 h might have been high enough to balance potential uptake (King et al. 2005; Nugroho and Frank 2011). Another potential explanation is that the effluent might trigger avoidance behavior to reduce the actual exposure (Hartmann et al. 2016). Avoidance was however not tested and not specifically noted upon visual inspection (e.g., prolonged valve closure), except for the 100 % effluent exposure in the preliminary experiment. Regardless, the overall implication, based on measured water and tissue concentrations, is that metal uptake was in most cases balanced or exceeded by excretion.

### Treatment effects

Stress proteins (here *hsp70*, *hsp90*, and *mt*) and markers of redox homeostasis (here *cat*, *gst*, GST, and *sod*) have been suggested as two key groups of biomarkers for general metal toxicity (Le Saux et al. 2020). Even low metal concentrations have been demonstrated to increase bivalve expression and activity of *cat*, *gst*, GST, *hsp70*, *mt*, and *sod* by ≥ 50 % (e.g., Ciacci et al. 2012; Franzellitti et al. 2020; Li et al. 2018; Perić and Burić 2019). In contrast, effect sizes from the effluent exposure were overall small. The mussel gill is the first organ in contact with waterborne pollutants, which may explain the responses in AChE and *hsp90*. However, apart from AChE and *hsp90*, all gill biomarker signals in the effluent exposure were within ± 12 % of the control. Hence, potential responses were not distinguishable from baseline noise. In digestive glands, all biomarkers responded to the effluent by ≤ 28 % increases, consistently non-significant despite elevated tissue levels of, e.g., Ni. One possible explanation could be a certain level of general metal tolerance, as the experimental mussels had previously been exposed to higher concentrations of, e.g., Fe, Mn, and Al (Table A.1, appendix A). Adaptation to metal exposure might for instance cause inter-population differences in transcriptional response patterns (Milan et al. 2016). This would affect the predictability of, e.g., biomarker effect sizes, potentially reducing the general sensitivity to relevant changes in the environment. Furthermore, it is possible that larger effect sizes would have been observed in immediate or long-term responses, but simply not captured by the 96-h static exposure. In order for a biomarker to be robust in, e.g., environmental monitoring, responses also require a certain degree of stability over time. Thus, results suggest that selected biomarkers, with potential exceptions of gill AChE and *hsp90*, were separately not robust and/or not sensitive enough to detect the effluent exposure in particular and perhaps not low to moderate stress in general.

AChE activity is quite commonly inhibited by chemical stressors (Bocquené and Galgani 1998). For instance, AChE inhibition has been demonstrated in *Anodonta cygnea*, a close relative to *A. anatina*, after acute exposure to low levels of a complex metal mixture (Butrimavičienė et al. 2019). While performed without replication of the effluent treatments, implication of AChE inhibition was also observed in our preliminary experiment, however, overlapping with the response range in the control group. In contrast, the main experiment demonstrated a clear 40 % increase in AChE gill activity in the effluent exposure. These seemingly contradictive results likely reflect high AChE variability and insufficient replication in the preliminary experiment. In fact, increased activity of AChE and other cholinesterases has been previously observed in other taxa after acute metal exposures (Brahma and Gupta 2020; Dahms-Verster et al. 2020; Oliva et al. 2019). Taken together, this suggests that AChE in *A. anatina* is quite variable, and that this enzyme might be less robust as a biomarker than what is often assumed.

Expression of *hsp90* demonstrated a treatment/sex interaction in gills. In this general stress marker, effluent exposure induced a 79–100% higher expression in both males and females but only compared to control females. This suggests that at least in gravid females, current stress levels were enough to induce a clearly detectable biomarker response. Still, it must be noted that both treatment groups consisted of 70–80% males, which for *hsp90* would obscure this effect if not including sex in the model. Therefore, without consideration of sex interactions, only a single biomarker (AChE in gills) showed a distinguishable treatment response under current exposure.

### Sex effects and response variability

Consistent with previous findings in *A. anatina* (Ekelund Ugge et al. 2020), we found higher *hsp70* expression in gills and lower AChE activity in digestive glands of gravid females, compared to males. In addition, the current study detected a sex effect in gill AChE activity as well as the *hsp90* treatment/sex interaction (discussed under the “Treatment effects” section), while there were no differences in, e.g., *cat*, *mt*, or GST as described previously (Ekelund Ugge et al. 2020). The different observations could result from random variation or differences between experiments (e.g., experiments carried out at different temperatures, mussels potentially collected or exposed at different stages of gravidness). Overall, the results therefore highlight biomarker variability, suggesting sex, and in particular gravidness, as potential confounding factors.

Responses to chemical stress may to a certain extent be buffered by various biological and ecological processes, and responses on one level often do not translate proportionally to adverse effects at higher organizational levels (e.g., Forbes and Calow 2002; Geist et al. 2007). Conversely, even when the mean response of an ecotoxicological endpoint remains unaffected by stress, underlying variation can potentially increase, and thus, response variability has in itself been suggested as a relevant toxicological endpoint (Nikinmaa and Anttila 2019). In addition to variability introduced by sex differences, we demonstrated an increase in variation for a majority of markers in *A. anatina* from the effluent exposure. In, e.g., risk assessment and environmental monitoring, biological responses to chemical stress should preferably be approached both by using multiple biomarkers (ideally in multiple tissues) and by incorporating variability measures in such biomarker panels.

## Conclusions

The small effect sizes suggest an inability of the chosen biomarkers to reliably indicate exposure to anthropogenic effluents in *A. anatina*. Only two biomarkers, one biochemical and one transcriptional, responded to exposure. Furthermore, despite increased tissue concentration of Ni in digestive glands, treatment responses were only observed in gills. This is further complicated by the confounding factor gravidness, which mainly appears to affect gill responses. An overall increase in variation across markers after the effluent exposure suggests that multi-biomarker approaches may potentially increase robustness for detection of chemical stress, despite (and potentially due to) high inherent variability of the separate markers. In future research, we propose continued assessment of multi-biomarker approaches as well as inter- and intra-population variability, both in terms of confounding effects and marker variation as a potential endpoint in itself.

## Supplementary information

ESM 1 (PDF 372 kb)

## Data Availability

Experimental datasets, as well as literature datasets used for toxic unit (TU) calculations, can be found at https://data.mendeley.com/datasets/jc469bc5mv/1.
