# Supplementary material for: Molecular biomarker responses in the freshwater mussel Anodonta anatina exposed to an industrial wastewater effluent
Source: Environ Sci Pollut Res Int. 2021 Aug 7;29(2):2158–70. doi: 10.1007/s11356-021-15633-4 (PMC8732836; doi:10.1007/s11356-021-15633-4)
Supplement: Supplementary file 1 — (PDF 372 kb) [file 11356_2021_15633_MOESM1_ESM.pdf]

Appendix A

Molecular biomarker responses in the freshwater mussel *Anodonta anatina* exposed to an industrial wastewater effluent

Gustaf MO Ekelund Ugge[a,b†], Annie Jonsson[b], Olof Berglund[a]

[a]Department of Biology, Lund University, Sölvegatan 37, 223 62 Lund, Sweden

[b]School of Bioscience, University of Skövde, Högskolevägen 3, 541 28 Skövde, Sweden

[†]Corresponding author at: Department of Biology, Lund University, Sölvegatan 37, 223 62 Lund, Sweden.

E-mail address: gustaf.ekelund_ugge@biol.lu.se

Appendix A

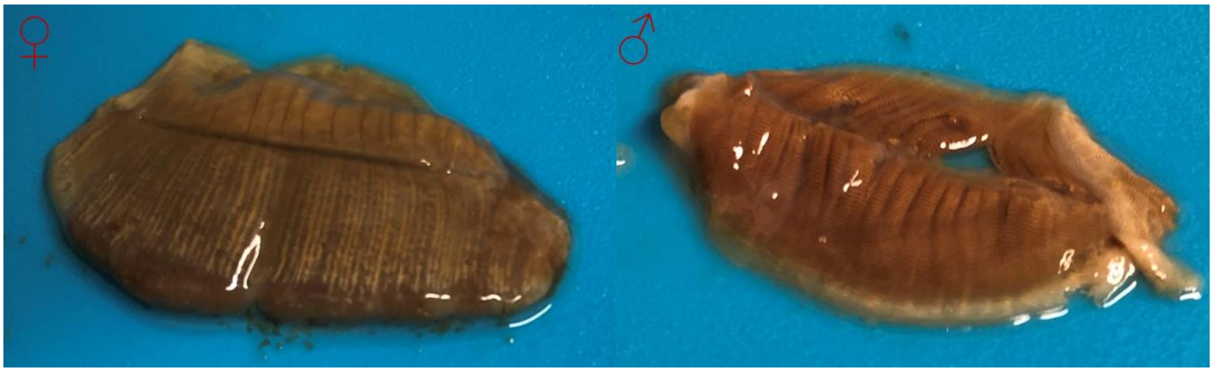

Figure A.1 Extracted gills of gravid (left) and non-gravid (right) *Anodonta anatina*.

Appendix A

Preliminary dose-finding experiment

Mussels for the preliminary experiment were collected the same date and at the same location as the main experiment mussels (19th of September 2018 in Vinne å, Southern Sweden). They were kept in a separate 40 L aquarium and acclimatized to laboratory conditions for 16 days. During acclimatization and experimental exposure, conditions were the same as in the main experiment.

For the experiment, effluent water was stepwise diluted with standardized freshwater into seven concentrations (4.7-100 %), using a dilution factor of $1\frac{2}{3}$. Mussels (length 69 ± 9 mm) were exposed for 96 h to effluent (*n* = 1 per concentration) or to a control treatment of standardized freshwater (*n* = 3), and gill AChE activities were assessed (see *Biomarker analysis* section under main article M&M).

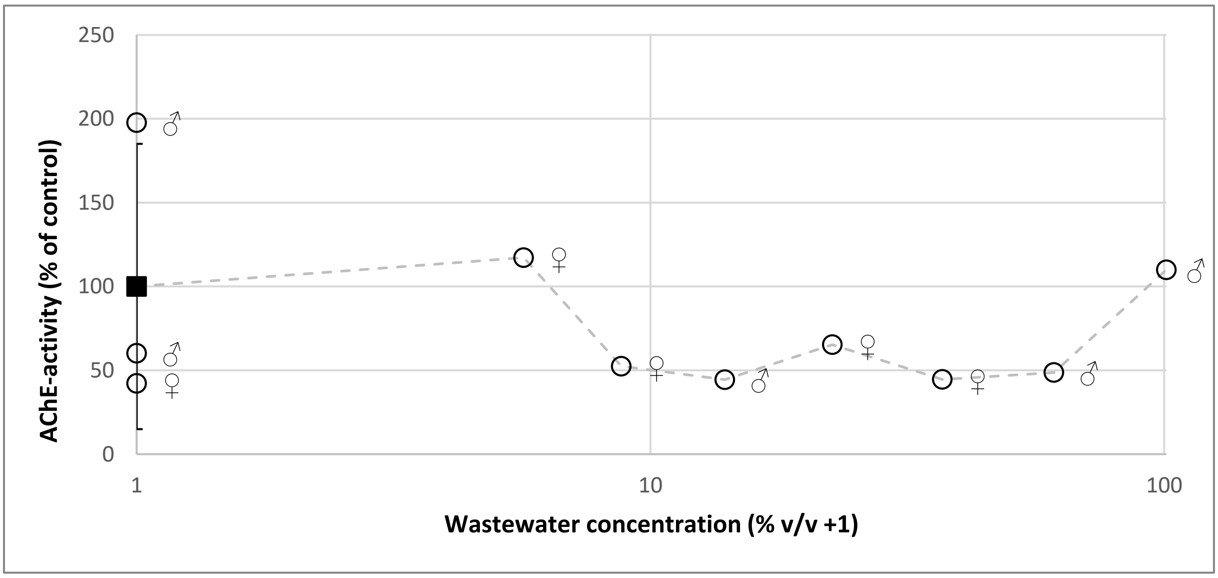

Figure A.2 Relative acetylcholinesterase (AChE) activity in gills of *Anodonta anatina* after 96 h exposure to industrial wastewater effluent. Results are part of a preliminary dose-finding experiment consisting of a control treatment (*n* = 3) and industrial wastewater in the range of 4.7 – 100 % (volume/volume) of the initial effluent concentration (*n* = 1 per treatment, in total seven concentrations). Circles represent individual mussels, while the black square and error bars show control group mean and SD. Symbols ♀ and ♂ denote gravid and non-gravid individuals.

Appendix A

Table A.1 Chemical composition of Vinne å water (*n* = 7). Water (unfiltered) was sampled between January and July 2018 (January 5th and 30th, February 21st, April 5th and 27th, May 27th and July 13th). † ≥ 1 sample <LOR.

Element	Median (µg/L)	Min – max (µg/L)
Ca	56 000	34 000 – 78 000
K	1 900	1 700 – 2 300
Mg	2 200	1 600 – 2 700
Na	11 000	8 500 – 15 000
Al	94	22 – 210
As	0.27	0.23 – 0.32
Ba	34	22 – 44
Cd	0.016	0.0033 – 0.034
Co	0.19	0.077 – 0.23
Cr	0.21	0.055 – 0.32
Cu	2.4	0.95 – 3.4
Fe	463	100 – 690
Hg	0.0035	0.002† – 0.0081
Mn	60	45 – 68
Mo	0.28	0.22 – 0.38
Ni	0.84	0.31 – 1.5
P	33	19 – 43
Pb	0.22	0.061 – 0.34
Si	3 800	2 800 – 4 400
Sr	120	77 – 180
V	1.0	0.81 – 1.2
Zn	4.0	0.85 – 5.1

Appendix A

Toxic unit (TU) estimations

Data based on eggs, embryos and larvae were excluded from each metal dataset, and so were entries with unclear effect concentrations (e.g. denoted by 'NR', '~', '<' or '>'). Juveniles were included only from studies that did not include adults. Consequently, if both adults and juveniles of the same species were examined in a single study, only the adult data were extracted. In studies reporting multiple effect concentrations for the same species (e.g. due to different exposure setups), all entries except the highest concentration were excluded in order not to overestimate the stressor. After the selection above, one entry for each species per study was used to identify the median mollusk 96 h EC_{50} and LC_{50} of each metal (Table A.2, full datasets and references presented in appendices B and C).

Appendix A

Table A.2 Metal toxic units in the control and wastewater treatments, and measured concentrations as fractions of respective environmental quality standard (EQS). Toxic units are based on mollusk 96 h 50 % effect concentration of sublethal organism effects (EC_{50}) and mortality (LC_{50}). Metal toxicity is assumed additive in the mixture toxic units (TU_M) of the control and wastewater exposures. Toxicity data were retrieved from the U.S. EPA ECOTOX database (appendices B and C).

	TU EC_{50}		Median 96 h EC_{50} (µg/L)	TU LC_{50}		Median 96 h LC_{50} (µg/L)	Fraction of EQS		EQS (annual average; µg/L)
Metal	Control	Wastewater	(# of EC_{50} entries)	Control	Wastewater	(# of LC_{50} entries)	Control	Wastewater	
Al	$1.0 * 10^{-2}$	$2.0 * 10^{-2}$	2 400 (1)	$4.4 * 10^{-4}$	$8.7 * 10^{-4}$	55 500 (5)	-	-	-
As	$5.5 * 10^{-7}$	$2.1 * 10^{-6}$	110 000 (1)	$2.5 * 10^{-6}$	$9.6 * 10^{-6}$	24 500 (3)	$1.2 * 10^{-1}$	$4.7 * 10^{-1}$	0.50[1]
Ba	-	-	NA (0)	-	-	NA (0)	-	-	-
Cd	$3.0 * 10^{-6}$	$1.3 * 10^{-5}$	1 700 (4)	$2.4 * 10^{-6}$	$1.0 * 10^{-5}$	2 200 (78)	$2.1 * 10^{-2}$	$9.0 * 10^{-2}$	0.25[1,2,3]
Co	-	-	NA (0)	-	-	NA (0)	-	-	-
Cr	-	-	NA (0)	$1.5 * 10^{-6}$	$2.0 * 10^{-4}$	15 200 (18)	$6.8 * 10^{-3}$	$9.0 * 10^{-1}$	3.4[1]
Cu	$9.2 * 10^{-3}$	$1.0 * 10^{-1}$	74 (13)	$2.2 * 10^{-3}$	$2.4 * 10^{-2}$	315 (112)	**1.4**	**15**	0.5[1,4]
Fe	$7.1 * 10^{-5}$	$4.4 * 10^{-4}$	64 (2)	$4.4 * 10^{-7}$	$2.7 * 10^{-6}$	10 300 (2)	-	-	-
Hg	$\leq 3.0 * 10^{-6}$	$\leq 3.0 * 10^{-6}$	670 (3)	$\leq 1 * 10^{-5}$	$\leq 1 * 10^{-5}$	200 (43)	-	-	-
Mn	-	-	NA (0)	$5.5 * 10^{-5}$	$1.4 * 10^{-4}$	45 600 (3)	-	-	-
Mo	-	-	NA (0)	-	-	NA (0)	-	-	-
Ni	$7.9 * 10^{-5}$	$2.1 * 10^{-2}$	770 (1)	$4.3 * 10^{-5}$	$1.1 * 10^{-2}$	1 440 (19)	$1.5 * 10^{-2}$	**4.1**	4[1,2,4]
Pb	-	-	NA (0)	$1.4 * 10^{-5}$	$1.1 * 10^{-5}$	10 300 (19)	$2.3 * 10^{-2}$	$9.2 * 10^{-2}$	1.2[1,2,4]
Sr	-	-	NA (0)	-	-	NA (0)	-	-	-
V	-	-	NA (0)	-	-	NA (0)	-	-	-
Zn	$5.4 * 10^{-4}$	$8.4 * 10^{-3}$	1 000 (5)	$1.2 * 10^{-4}$	$1.9 * 10^{-3}$	4 510 (61)	$9.8 * 10^{-2}$	**1.5**	5.5[1,4]
**TU_M**	**$2.0 * 10^{-2}$**	**$1.5 * 10^{-1}$**		**$2.8 * 10^{-3}$**	**$3.9 * 10^{-2}$**				

[1]Havs- och vattenmyndigheten 2019
[2]European parliament and council 2013
[3]Based on water hardness ≥ 200 mg/L $CaCO_3$
[4]Bioavailable concentration

Table A.3 Primer sequences used in the RT-qPCR assays. Assay details are presented in Ekelund Ugge *et al*. 2020.

Gene	Sequence (5’-3’) Forward	Reverse
β-actin (*β-act*)	CAAACTGGGATGATATGGA	CATCTTTTCTCTGTTGGC
28S rRNA (28S)	ATCCTTGCTCGTCACGAC	GTACCAACCCTTCCTACG
Catalase (*cat*)	GGAAGACTGACCAGGGTAT	CCTCAGCGATGGCATTGTA
Glutathione-S-transferase (*gst*)	GTCCAACACCATGCTGAG	GTAGTCCTCCACTCCATCAT
Heat shock protein 70 (*hsp70*)	GGTATTGAGACGGCTGGT	CACACCAGGCTGGTTGTC
Heat shock protein 90 (*hsp90*)	TACCATTGCCAAATCTGG	ACACCAAACTGCCCAATCA
Metallothionein (*mt*)	ATGCAACTGCCTTGAGAC	ACTTTACATCCAGGACACTT
Superoxide dismutase (*sod*)	GCTACGGTCATTCCACTCT	CCAGTTATCTCACCAGTTATGTTC

Appendix A

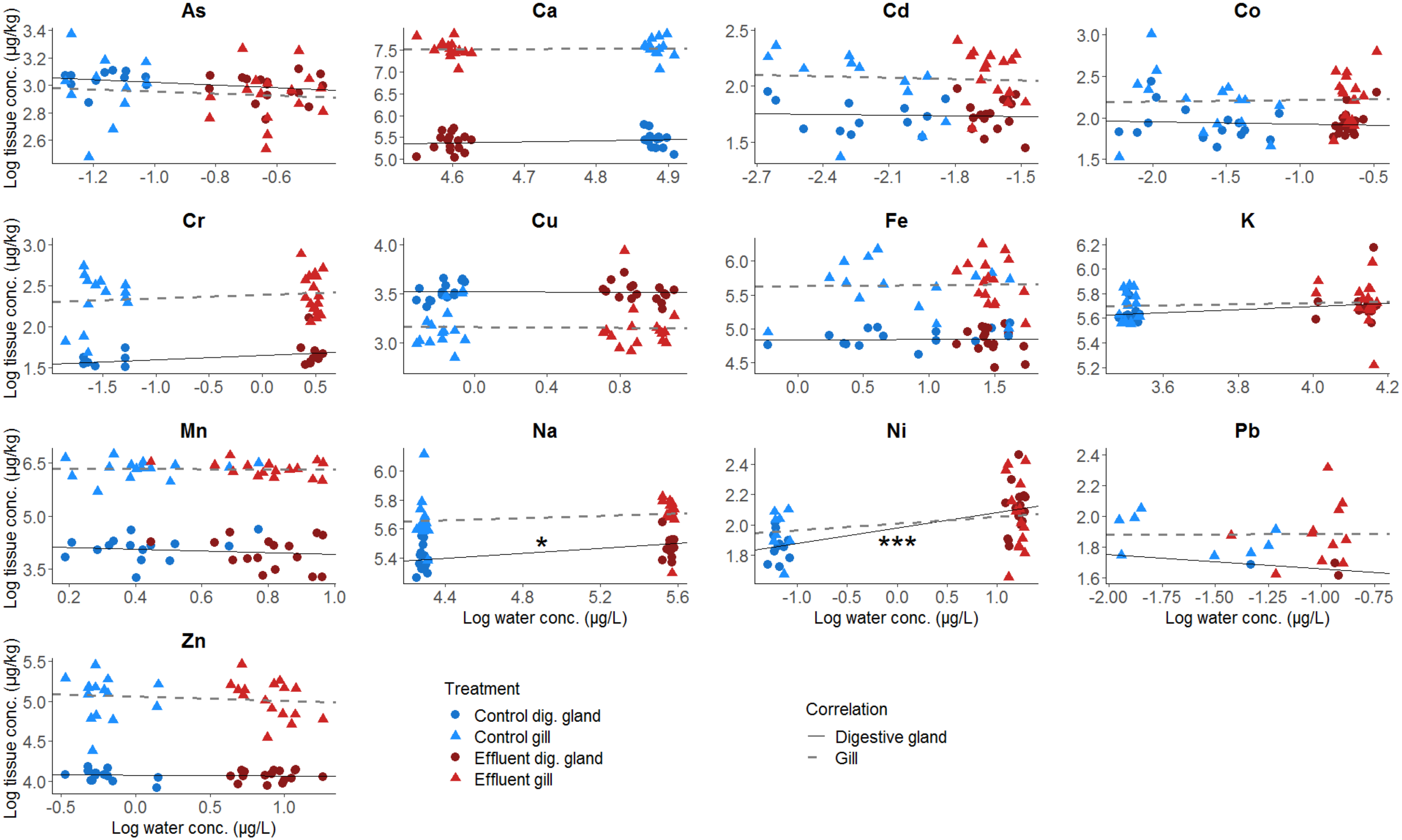

Figure A.3 Tissue and water concentrations (μg/kg WW and μg/L, respectively) and their correlation in *Anodonta anatina* experimentally exposed to industrial effluent water for 96 h. Significance of correlations is implied by * = p<0.05, ** = p<0.01 and *** = p<0.001.

Table A.4 Correlation between tissue and ambient water concentration in *A. anatina* experimentally exposed to industrial effluent water. For the correlation analysis, any sample was excluded for which measured water and/or tissue concentration was below the level of reporting (LOR). Hg was analyzed in both tissue and water samples, but all water samples showed levels <LOR and were thus excluded.

	Digestive gland			Gill		
Element	Corr. coeff. (r)	Significance (p)	Slope (k)	Corr. coeff. (r)	Significance (p)	Slope (k)
As	-0.318	0.106	-0.0994	-0.106	0.599	-0.0777
Ca	0.169	0.363	0.225	0.0416	0.824	0.0530
Cd	-0.0574	0.772	-0.0229	-0.0577	0.771	-0.0430
Co	-0.0771	0.680	-0.0246	0.0295	0.875	0.0172
Cr	0.399	0.0811	0.0525	0.160	0.400	0.0436
Cu	-0.0333	0.859	-0.00526	-0.0232	0.901	-0.00901
Fe	0.0151	0.936	0.00472	0.0240	0.898	0.0162
Hg	-	-	-	-	-	-
K	0.336	0.0645	0.121	0.104	0.578	0.0487
Mn	-0.156	0.401	-0.258	-0.0454	0.808	-0.0440
Na	0.527	***0.00230***	0.0853	0.181	0.329	0.0384
Ni	0.685	***8.20 *10^{-5}***	0.101	0.242	0.279	0.0433
Pb	-0.474	0.686	-0.0912	0.00705	0.977	0.00307
Zn	-0.0999	0.593	-0.0112	-0.118	0.528	-0.0494

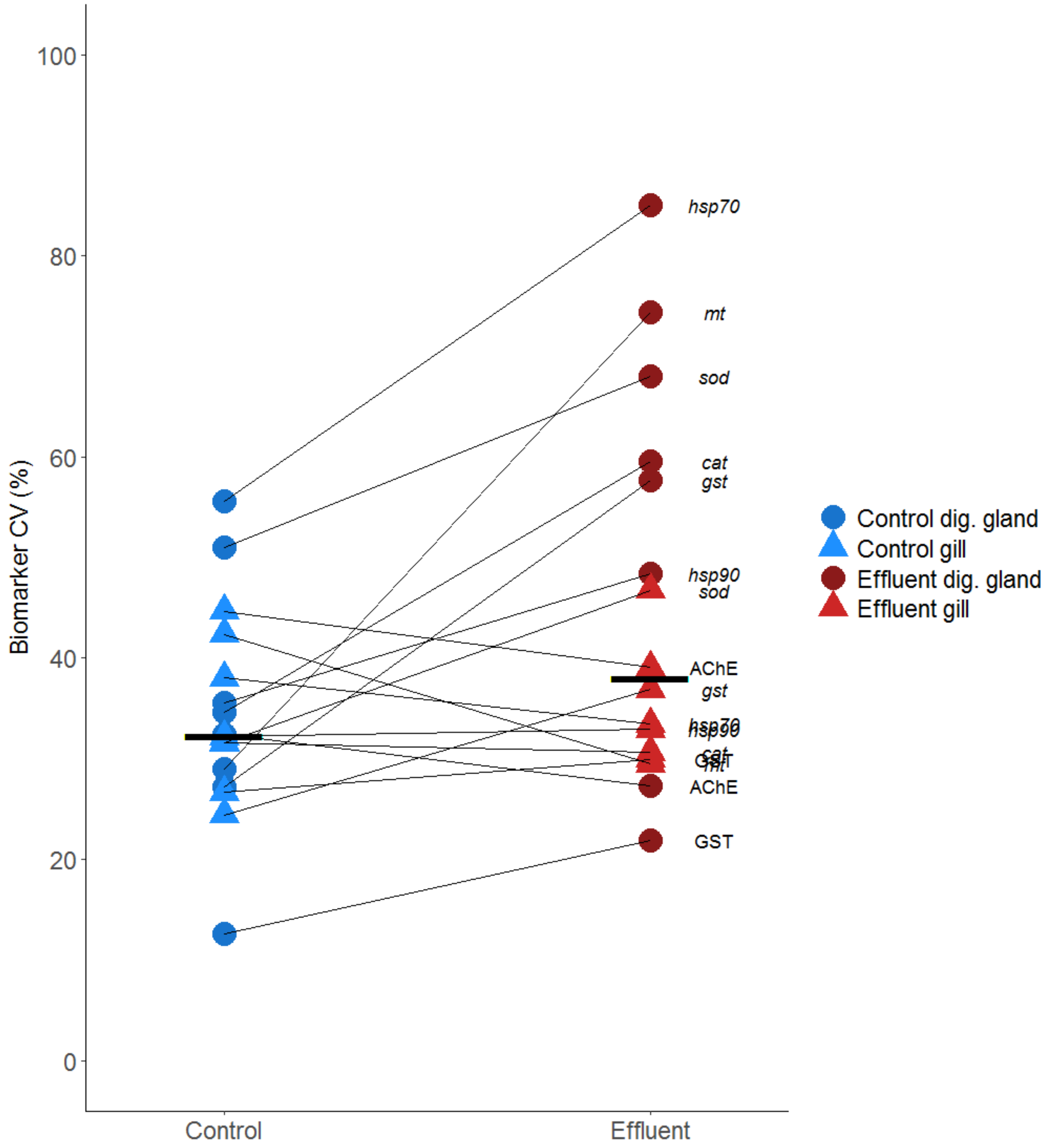

Figure A.4 The coefficient of variation (CV) across biomarkers and treatments. Bars show median CV.
